# A new tissue segmentation method to calculate 3D dose in small animal radiation therapy

**DOI:** 10.1186/s13014-018-0971-8

**Published:** 2018-02-26

**Authors:** C. Noblet, G. Delpon, S. Supiot, V. Potiron, F. Paris, S. Chiavassa

**Affiliations:** 1 Medical Physics Department, Institut de Cancérologie de l’Ouest Centre René Gauducheau, 44805 Saint-Herblain, France; 2 CRCINA, Inserm U1232, Université de Nantes, Nantes, France; 3 Radiotherapy Department, Institut de Cancérologie de l’Ouest Centre René Gauducheau, 44805 Saint-Herblain, France; 4 LABCT, Institut de Cancérologie de l’Ouest Centre René Gauducheau, 44805 Saint-Herblain, France

**Keywords:** Monte Carlo simulations, Pre-clinical radiation therapy, Tissue segmentation

## Abstract

**Background:**

In pre-clinical animal experiments, radiation delivery is usually delivered with kV photon beams, in contrast to the MV beams used in clinical irradiation, because of the small size of the animals. At this medium energy range, however, the contribution of the photoelectric effect to absorbed dose is significant. Accurate dose calculation therefore requires a more detailed tissue definition because both density (ρ) and elemental composition (Z_eff_) affect the dose distribution. Moreover, when applied to cone beam CT (CBCT) acquisitions, the stoichiometric calibration of HU becomes inefficient as it is designed for highly collimated fan beam CT acquisitions. In this study, we propose an automatic tissue segmentation method of CBCT imaging that assigns both density (ρ) and elemental composition (Z_eff_) in small animal dose calculation.

**Methods:**

The method is based on the relationship found between CBCT number and ρ*Z_eff_ product computed from known materials. Monte Carlo calculations were performed to evaluate the impact of ρZ_eff_ variation on the absorbed dose in tissues. These results led to the creation of a tissue database composed of artificial tissues interpolated from tissue values published by the ICRU. The ρZ_eff_ method was validated by measuring transmitted doses through tissue substitute cylinders and a mouse with EBT3 film. Measurements were compared to the results of the Monte Carlo calculations.

**Results:**

The study of the impact of ρZ_eff_ variation over the range of materials, from ρZ_eff_ = 2 g.cm^− 3^ (lung) to 27 g.cm^− 3^ (cortical bone) led to the creation of 125 artificial tissues. For tissue substitute cylinders, the use of ρZ_eff_ method led to maximal and average relative differences between the Monte Carlo results and the EBT3 measurements of 3.6% and 1.6%. Equivalent comparison for the mouse gave maximal and average relative differences of 4.4% and 1.2%, inside the 80% isodose area. Gamma analysis led to a 94.9% success rate in the 10% isodose area with 4% and 0.3 mm criteria in dose and distance.

**Conclusions:**

Our new tissue segmentation method was developed for 40kVp CBCT images. Both density and elemental composition are assigned to each voxel by using a relationship between HU and the product ρZ_eff_. The method, validated by comparing measurements and calculations, enables more accurate small animal dose distribution calculated on low energy CBCT images.

## Background

Over the past few years, pre-clinical radiation therapy devices dedicated to small animals have been widely developed to reliably transpose clinical techniques to small animals [1, 2]. Photon beam energy was reduced to 100-400kVp to adapt beam penetration and penumbra to the size of small animals (essentially mice and rats) and to allow the use of very small beams, as narrow as 1 mm in diameter. However, this medium energy range leads to a higher proportion of photoelectric effect in small animals than observed in the MV energy range in human patients. Absorbed dose continues to depend significantly on mass density, but also on elemental composition, as the photoelectric cross-section depends on the ${Z}_{eff}^{3-4}$ (effective atomic number) [3, 4].

In this context the analytical algorithms used to estimate absorbed dose in clinical practice at MV energy range are no longer valid. Monte Carlo methods remain the best alternative for the accurate calculation of 3D absorbed dose distributions in small animals. An accurate knowledge of tissue elemental composition is necessary to achieve a dose precision level equivalent to that of clinical practice (1–3%) at medium energy range. The tissue distribution is basically estimated from computed tomography (CT) images. Tissue equivalent materials of known densities are scanned to obtain a CT number to mass or electronic density conversion curve. In clinical practice, such a conversion curve suffices since at MV energy range the Compton effect predominates, and it essentially depends on material density. Unfortunately, density alone is insufficient to define tissues at medium energy ranges. Two tissues with the same density but different effective atomic numbers may potentially receive significantly different absorbed doses [5]. The elemental composition of materials is therefore required.

In this study, we describe an original automatic tissue segmentation method for the calculation of absorbed dose in the context of small animal radiation therapy.

## Methods

### HU calculation applied to acquisitions with a 40kVp uncollimated cone beam

#### Stoichiometric method

To automatically obtain the elemental composition of tissues from CT images, a stoichiometric calibration method [6] was originally proposed by Schneider et al. [7]. It relies on CT scanning known materials to find a relationship between CT number, physical density and effective atomic number by fitting the parameters of the Jackson and Hawkes equation (Eq. 1) [8, 9].

1 $$ \mu ={\rho N}_A{\sum}_{i=1}^n\left(\frac{w_i}{A_i}\left({K}^{\mathrm{KN}}{Z}_i+{K}^{\mathrm{ph}}{Z}_i^{4.62}+{K}^{\mathrm{sca}}{Z}_i^{2.86}\right)\right) $$

$$ CTnumber=1000\ast \left(\frac{\mu }{\mu_{water}}-1\right) $$

μ = attenuation coefficient.

i = chemical element.

w = elemental weight.

ρ = mass density.

N_A_ = Avogadro constant.

A_i_ = mass number of i.

Z_i_ = atomic number of i.

K^KN^ = Klein-Nishina coefficient.

K^ph^ = constant characterizing the photoelectric absorption.

K^sca^ = constant characterizing the cross section of the coherent and incoherent scattering.

In our institution, small animal images are performed using the cone-beam CT (CBCT) scan integrated in the XRAD225Cx preclinical irradiator (Precision X-Ray Inc., CT, USA) using a non-collimated beam, rather than with a CT scan using a collimated fan beam. Eleven substitute tissue materials of known densities and elemental compositions (Gammex-RMI, WI, USA) were scanned with the CBCT imager at 40kVp to evaluate the stoichiometric method in our geometric settings. CBCT numbers were also calculated with Eq. 1 and compared to the experimental results.

#### ρZ_eff_ segmentation method

**Fig. 1:**
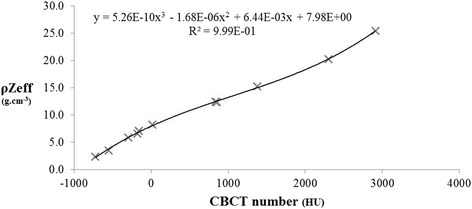
CBCT number variation with the product of physical density ρ and effective atomic number Z_eff_. Each x represents a different insert from the Gammex-RMI phantom. The solid line represents the third degree polynomial fitting curve

At low and medium energy range, any accurate tissue segmentation method must take into account both density (ρ) and elemental composition, so the relationship between the CBCT number, ρ and Z_eff_ must be determined. CBCT images previously acquired from the 11 substitute tissue materials were used to test different plots of a function of ρ and Z_eff_ versus CBCT number. The curve ρZ_eff_ versus CBCT number led to a successful monotonic relationship (Fig. 1).

Using the set of substitute tissue materials from the Gammex-RMI phantom, a third degree polynomial equation fitted the relationship of ρZ_eff_ versus CBCT number very well, with a 0.999 correlation coefficient. Based on this (ρZ_eff_, HU) relationship, a CBCT number can be calculated for every material of known ρZ_eff_, and reciprocally.

The proposed ρZ_eff_ assignation can be applied as follows:

i) a 40kVp CBCT scan of materials with known ρZ_eff_ is performed;
ii) the (HU, ρZ_eff_) polynomial relationship is determined;
iii) the HU for a list of tissues of known ρZ_eff_ are calculated;
iv) tissues are assigned to CBCT images based on the list in iii).

### “Dose-equivalent” tissue calculation

To obtain the expected dose precision level (1–3%) with this “ρZ_eff_” method, tissues must be segmented in such a way that the dose difference between two neighboring tissues (in terms of ρZ_eff_) is lower than 2–3%. In other words, we need a list of tissues generated with an adjusted ρZ_eff_ step.

Absorbed dose was calculated in different tissues of known ρZ_eff_ with a validated GATEv7 Monte Carlo model [10, 11] as follows: tissues from ICRU report 44 [12] and 46 [13] (Table 1) were attributed to a piece of 5x5cm^2^ and 0.5 cm thick tissue inserted at 1.5 cm depth in a 5x5x5cm^3^ water tank. A 5 mm diameter circular beam of 225kVp was simulated. Absorbed dose in tissue was normalized to absorbed dose at the same position in a homogeneous water tank. These calculated absorbed doses were used to estimate the maximum ρZ_eff_ difference between tissues required to reach a 2% dose precision. A list of tissues with a ρZ_eff_ step corresponding to this maximum difference was generated. The HU was calculated for each tissue using the (HU, ρZ_eff_) polynomial relationship.

**Table 1:** Tissues from ICRU report 44 [12] and 46 [13]

ICRU tissues	Physical density ρ (g.cm^3^)	Z_eff_	ρZ_eff_
Lung inflated	0.26	7.88	2.05
Adipose	0.95	6.67	6.34
Yellow marrow	0.98	6.56	6.43
Average soft tissue adult female	1.02	7.44	7.59
Red marrow	1.03	7.44	7.66
Water	1.00	7.73	7.73
Average soft tissue adult male	1.03	7.60	7.83
GI tract	1.03	7.71	7.94
Pancreas	1.04	7.70	8.01
Breast	1.02	7.88	8.04
Eyes lens	1.07	7.54	8.07
Lymph	1.03	7.84	8.07
Testis	1.04	7.82	8.13
Brain	1.04	7.88	8.19
Urinary bladder filled	1.03	7.98	8.22
Kidney	1.05	7.84	8.23
Ovary	1.05	7.84	8.24
Muscle	1.05	7.85	8.24
Lung deflated	1.05	7.88	8.27
Skin	1.09	7.63	8.31
Liver	1.06	7.87	8.34
Spleen	1.06	7.87	8.34
Heart	1.06	7.95	8.43
Blood	1.06	7.97	8.45
Cartillage	1.10	8.33	9.16
Thyroid	1.05	9.19	9.65
Spongiosa	1.18	10.74	12.68
Sacrum	1.29	11.46	14.79
Femur	1.33	12.09	16.08
Humerus	1.46	12.61	18.41
Cranium	1.61	13.13	21.14
Mandible	1.68	13.33	22.40
Cortical bone 5 year Child	1.75	13.56	23.72
Cortical bone	1.92	13.98	26.84

### Validation of the tissue assignation method

#### Transmitted dose through known materials

**Fig. 2:**
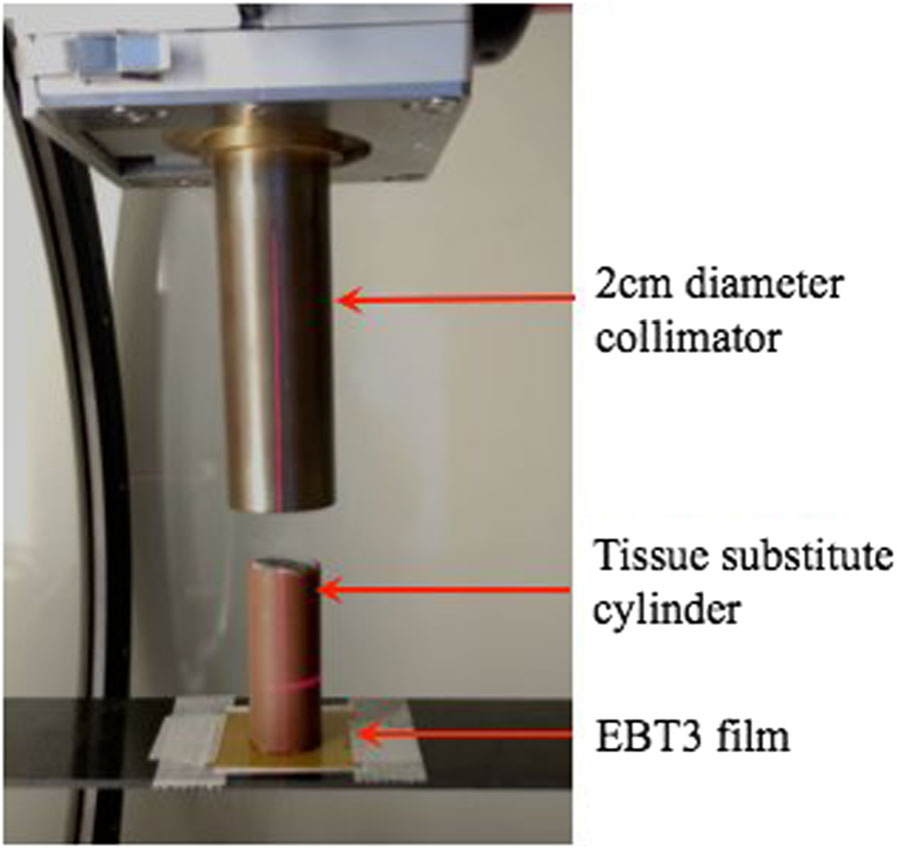
Transmitted dose through the tissue substitute cylinders measured with an EBT3 film. Five minutes of irradiation were performed with an anterior 2 cm diameter beam at 225kVp

In each voxel, the tissue assignment is automatically performed using the previously obtained tissue database. For each of these tissues, an HU interval has been calculated with the (ρZ_eff_, HU) polynomial relationship illustrated in Fig. 1. However, HU noise in acquired images (up to 30HU) and polynomial relationship can introduce bias, and lead to incorrect tissue assignment. In order to estimate the accuracy of our segmentation method, the dose transmitted through tissue-equivalent cylinders (Gammex-RMI, WI, USA) was measured. Indeed, the measurement of absorbed dose within a medium cannot be performed as detectors are usually calibrated in terms of dose to water and thus provide absorbed dose to water in medium. Consequently, the validation was based on transmitted doses measured with EBT3 Gafchromic films (International Specialty Products, NJ, USA). EBT3 films were positioned under the tissue-equivalent cylinders at 33 cm from the source (Fig. 2). The same irradiation procedure was then simulated with our MC model with two different settings:

i. MC simulations were performed by assigning the manufacturer’s published compositions for the cylinders.
ii. Artificial tissues were assigned to each voxel based on our automatic segmentation method and a CBCT number obtained for each cylinder at 40kVp. The high density cylinders exhibited a significant beam hardening effect in the CBCT scan, so it was decided to assign materials according to the CBCT number obtained at the periphery of the cylinder. This effect is very limited in preclinical radiation therapy with small animals, as bone size is smaller than a few mm.

#### Transmitted dose through a mouse

An EBT3 film was positioned under the mouse to be irradiated with an anterior 2 cm diameter beam at 225kVp. A CBCT scan of the mouse was performed with the EBT3 in place to ensure that the irradiation was identical with our MC model, and avoid displacement of the mouse between scanning and irradiation, which could skew the MC/EBT3 result concordance. The location of the film in the CBCT scan could then also be used to derive the absorbed dose at the same position. Four 2 mm diameter radio-opaque balls were positioned near the edge of the film in order to facilitate registration between measurement and simulation.

In order to subtract the dose contribution due to the CBCT scan, the procedure was repeated with a second film positioned under the mouse and then scanned at 40kVp but not irradiated at 225kVp. The same calibration procedure to measure 2D absorbed dose with radiochromic film was applied to both films [10]. The EBT3 films were calibrated at 225kVp in 2 cm deep water at the isocenter with a 10 cm square beam. The reference dose was calculated at the same position according to the TRS-398 dosimetric protocol [14–16] published by the International Atomic Energy Agency. Films were digitized 24 h after their irradiation with a V700 Epson scanner (Epson America Inc., CA, USA) at 200dpi resolution using three 16-bit monochrome channels to yield a Tiff image. Optical density was converted into grayscale values based on the triple channel analysis method [17, 18].

In the MC simulation, the EBT3’s properties were considered to be equivalent to those of water, as they were calibrated according to absorbed dose in water. The ρZ_eff_ method was applied at each voxel representing the mouse. Simulation output resolution was set to 0.2 mm × 0.2 mm × 0.2 mm, a trade-off between acceptable resolution and calculation time.

To compare the MC result with EBT3 measurements, a gamma analysis [19] was performed with RIT113 software (Radiological Image Technology Inc., CO, USA). The EBT3 measurement was set as the reference image and the MC result as the target image. Both were normalized to the same value. We evaluated the uncertainties and dimensions for acceptable gamma criteria in a previous study [10]. The dose difference (DD) criterion was set to 4% given that the measurement uncertainty was 3.2% and the statistical uncertainty of the MC simulations was less than 1.5%. Distance to agreement (DTA) was adapted from human to mouse according to image resolution (from 2x2x2mm^3^ to 0.2 × 0.2 × 0.2 mm^3^) and beam size (cm to mm). It was set to 0.3 mm.

## Results

### HU calculation applied to acquisitions with a 40kVp uncollimated cone beam

**Fig. 3:**
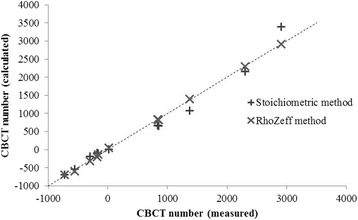
Measured versus calculated CBCT values with the stoichiometric and the ρZ_eff_ assignment methods

The stoichiometric method obtained differences greater than 100HU and up to 480HU between calculated and experimental CBCT numbers, especially for materials assimilated to bony tissues (Fig. 3).

Using the polynomial fitting equation of Fig. 1, measured and calculated CBCT numbers were in good agreement for all materials, with differences of less than 40HU (Fig. 3).

### “Dose-equivalent” tissue calculation

**Fig. 4:**
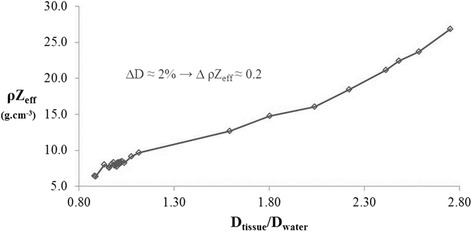
ρZ_eff_ variation with absorbed dose in ICRU tissues (see Table 1). MC dose calculations were performed for an anterior 5 mm 225kVp beam in a 5x5x5cm^3^ water tank with a 5 mm thick tissue insert at 1.5 cm depth in water. Absorbed dose in tissue was normalized to absorbed dose at the same position in the homogeneous water tank. This plot shows that a 0.2 ρZ_eff_ interval between two neighboring tissues is required to reach approximately 2% dose calculation precision

Figure 4 shows the absorbed dose in ICRU tissues versus ρZ_eff_. The ρZ_eff_ difference between tissues must be on average no larger than 0.2 g.cm^− 3^ to obtain a less than 2% dose difference. Unfortunately, the ρZ_eff_ difference is larger than 0.2 g.cm^− 3^ for many tissues defined in ICRU reports with ρZ_eff_ values higher than 10 g.cm^− 3^. In order to limit the dose difference to a maximum of 2% between two neighboring tissues, 125 materials were linearly interpolated based on the ρZ_eff_ values of ICRU tissues.

The linear interpolation performed for ρZ_eff_ values varied from 2 (ICRU inflated lung) up to 27 (ICRU cortical bone) in steps of 0.2. The elemental composition of each artificial tissue corresponded to a linear combination of the two nearest ICRU tissue neighbors.

For each of the 125 artificially created tissues for a specific ρZ_eff_ (between 2 and 27 in steps of 0.2):

i). The Z_eff_ of the tissue is linearly interpolated based on the known ρZ_eff_ values of the nearest ICRU tissues.
ii). The mass fraction w_i_ of each element i is a linear interpolation of elemental mass fractions of the two nearest existing ICRU tissues (neighbors in term of ρZ_eff_).
iii). The effective atomic number Z_eff_ is recalculated based on the interpolated w_i_ of each element.
iv). If the recalculated Z_eff_ differed by more than 5% from the expected Z_eff_ value in (i), the selected weight of element i w_i_ was iteratively increased or decreased by a 0.001 step depending on whether the difference was either positive or negative:
  о for ρZ_eff_ < 10: carbon and oxygen.
  о for ρZ_eff_ > 10: calcium and phosphor.

v). The mass fraction sum of all elements was recalculated. This must be equal to 1, or the hydrogen mass fraction is modified, given that hydrogen’s atomic number (Z = 1) has a negligible impact on Z_eff_ value.
vi). ρ is deducted from the expected value of ρZ_eff_ and the calculated value of Z_eff_.

There is no anatomical meaning in the definition of these materials. These artificial materials should be considered as “dose-equivalent” tissues with properties that lead to the same absorbed dose, rather than actual tissues of unknown composition. A corresponding CT number range was calculated with the (ρZ_eff_, HU) relationship for each of these 125 artificial tissues.

In summary, the reference ICRU tissues were only used to determine the maximum ρZ_eff_ difference needed between two neighboring tissues in order to obtain an absorbed dose precision in animals of less than 2% (Fig. 4), and to interpolate 125 artificial tissues with ρZ_eff_ values from 2 g.cm^− 3^ up to 27 g.cm^− 3^ in steps of 0.2, as determined by these obtained ρZ_eff_ differences.

### Validation of the tissue assignation method

#### Transmitted dose through known materials

The use of the manufacturer’s data in the MC computations yielded good agreement between the MC results and EBT3 transmission measurements (Table 2). The maximum relative difference was found to be 3% in all materials. The mean relative difference of exit absorbed doses was less than 1.5%. Those results confirmed the ability of our MC model and our film dosimetry method to calculate absorbed doses with good accuracy whatever the material. Our assignment method based on the (HU, ρZ_eff_) relationship, with no a priori knowledge of the material, then found transmitted absorbed doses to be in good agreement with EBT3 measurements. Maximum difference was 3.6%. The mean relative difference of exit absorbed doses was about 1.6% (Table 2).

**Table 2:** Transmitted dose through tissue substitute materials, measured and simulated with manufacturer’s data (Gammex) and the ρZ_eff_ tissue assignment method. EBT3 measurement uncertainty was 3.2% [10]

		Manufacturer’s data				ρZeff based tissue assignation method			
Tissue substitutes	EBT3 (Gy)	MC dose (Gy)	Stat. Uncert.	MC/EBT3 relative difference	MC/EBT3 absolute difference (Gy)	MC Dose (Gy)	Stat. Uncert.	MC/EBT3 relative difference	MC/EBT3 absolute difference (Gy)
CT solid water	4.58	4.61	1.4%	0.5%	0.03	4.51	1.3%	−1.5%	−0.07
Inner bone	3.44	3.50	1.5%	1.7%	0.06	3.41	1.5%	−1.0%	−0.03
Cortical bone	1.40	1.38	2.0%	−1.8%	−0.02	1.42	2.1%	1.3%	0.02
CB2 50%	1.99	1.93	1.8%	−3.0%	−0.06	2.02	1.8%	1.5%	0.03
CB2 30%	2.73	2.72	1.6%	−0.4%	−0.01	2.80	1.6%	2.6%	0.07
Breast	4.93	4.91	1.3%	−0.4%	−0.02	4.75	1.3%	−3.6%	−0.18
B200-bone	3.41	3.49	1.5%	2.4%	0.08	3.45	1.5%	1.1%	0.04
Adipose	5.29	5.35	1.3%	1.3%	0.07	5.29	1.3%	0.1%	0.01

#### Transmitted dose through a mouse

**Fig. 5:**
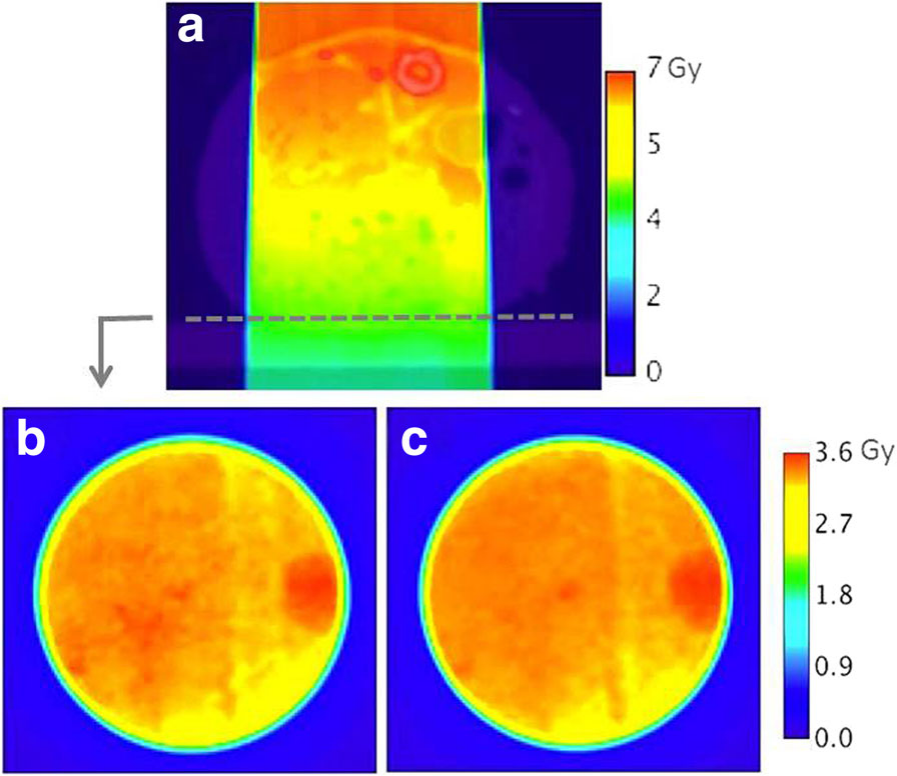
**a** Absorbed dose distribution in mouse (axial section). Transmitted dose through the mouse measured in coronal plane with **b** EBT3 film and **c** computed by MC calculations. EBT3 uncertainty was 3.2%. MC statistical uncertainty was lower than 1.5%

**Fig. 6:**
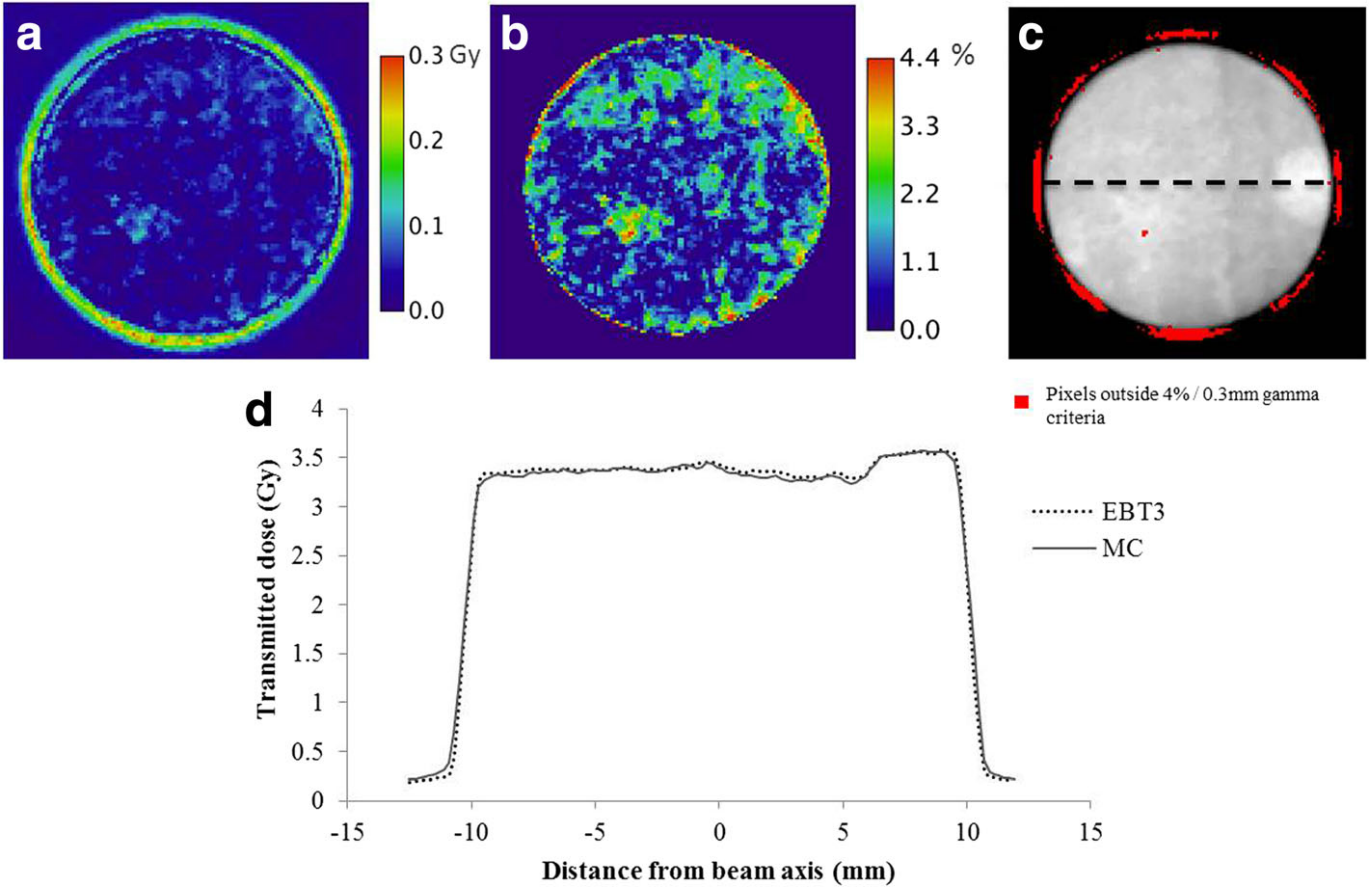
**a** Absorbed dose difference (Gy) between EBT3 and MC results. **b** Relative dose difference (%) between EBT3 and MC results performed inside the 80% isodose area. **c** Gamma analysis performed with 4% DD and 0.3 mm DTA, revealing a success rate of 94.9%. **d** Measured and calculated horizontal dose profiles along a diameter plotted with the dashed line on (**c**)

Measured absorbed dose in the EBT3 film was compared to the same coronal plane extracted from the MC 3D dose distribution (Fig. 5). EBT3 measurements and MC results agreed very well, with dose differences less than 0.3Gy, mainly in the field periphery (Fig. 6). Inside the 80% isodose area (the area where absorbed dose is more than 80% of the maximum dose) a 4.4% maximum and a 1.2% mean relative differences were found, corresponding to a 0.04Gy maximum dose difference. Gamma analysis revealed a 94.9% success rate in the 10% isodose area with 4% DD and 0.3 mm DTA criteria. Failed pixels were mainly localized in the penumbra where dose gradient was high. However, a profile line along a diameter (Fig. 6d) showed these discrepancies were slight.

## Discussion

In small animal radiotherapy practice, a limited number of tissues are generally assigned, such as air, lung, muscle and cortical bone, using differences in CT numbers. However, many studies have showed that this method leads to tissue mis-assignment and potentially large dosimetric errors. Chow et al. [3] calculated up to 400% deviation at 225kVp between absorbed dose calculated in a homogeneous mouse and in a mouse in which bone was considered. Verhaegen et al. [20] highlighted that an incorrect tissue assignment could lead to dose error greater than 40% at 250kVp whereas less than 10% error was found at 6MV and 15MV. Zhou et al. [21] have demonstrated that 47 different bony tissues were needed with a 120kVp beam to reach 2% dose accuracy. Bazalova et al. [4] suggest that 92 tissues must be defined to obtain 2% accuracy for dose calculation at 225 kVp, showing that a simplistic 4–8 tissue assignment could lead to differences of more than 30% when compared to a dose distribution calculated with 39 tissues. Those previous studies showed a large number of materials must be defined to reach a satisfactory dose accuracy in the range of 1–3%.

Our tissue segmentation method principle essentially follows the stoichiometric calibration method, fully described by Vanderstraeten et al. [6]. This method has particularly found application in proton therapy, where material attribution is also a critical parameter [22, 23], but suffers significant error in HU calculations based on CBCT acquisitions. The stoichiometric calibration was originally designed for a highly collimated fan beam [8], but it fails when applied to CBCT acquisitions whose divergent broad beam produces more scattered radiation. No gold standard tissue segmentation method exists for CBCT images and various approaches have been explored, such as, recently, dual energy CBCT [24]. Our method, based on the (HU, ρZ_eff_) relationship, showed satisfactory results for dose calculation at 225 kV based on 40kVp CBCT images. It was validated by measuring transmitted doses through known materials and unknown materials using respectively tissue-equivalent cylinders and a real mouse.

Elemental composition and densities for small animal tissues are still unknown. In the absence of data on small animal tissue composition, the use of human tissue to assign values for small animals is inevitable but remains questionable. However, the use of indirect segmentation methods, such as the stoichiometric or ρZ_eff_ methods allows the concept of “dose-equivalent” tissues to be defined. These assigned tissues are not anatomically consistent: they are artificially generated with the aim of being sufficiently discriminating between small animal tissues to provide accurate dose calculation.

## Conclusion

The major dosimetric impact of heterogeneities in small animal tissues means that tissue assignment is a primordial parameter for attaining reliable dose distributions for absorbed dose calculation in pre-clinical practice. We have shown that an automatic voxel by voxel tissue assignment method based on a third degree polynomial relationship between CT numbers (HU) and ρZ_eff_ is effective at reducing the dose distribution errors probable with simpler tissue assignment methods. Dose differences of less than 4% were found between measured and calculated dose transmitted though several tissue substitute materials with this new tissue attribution method. Less than 4.4% dose difference was obtained inside the 80% isodose area between measured and simulated dose transmitted through a mouse, suggesting satisfactory tissue assignment.

## Abbreviations

CBCT: Cone Beam Computed Tomography
CT: Computed Tomography
HU: Hounsfield unit
MC: Monte Carlo
Z_eff_: effective atomic number
ρ: mass density
